# Temporomandibular Joint Ganglion Cyst Causing Dynamic External Auditory Canal Obstruction and Position-Dependent Hearing Loss: A Case Report and Literature Review

**DOI:** 10.3390/life16050839

**Published:** 2026-05-19

**Authors:** Ali Akbar, Abdulrahman Meerza, Craig Pearl

**Affiliations:** 1 Katz Department of Oral and Maxillofacial Surgery, School of Dentistry, The University of Texas Health Science Center at Houston, Houston, TX 77054, USA; 2 Department of Surgical Sciences, College of Dentistry, Health Sciences Center, Kuwait University, Kuwait City 13060, Kuwait; 3 Department of Oral and Maxillofacial Surgery, Memorial Hermann Hospital, Texas Medical Center, Houston, TX 77030, USA

**Keywords:** temporomandibular joint, ganglion cyst, synovial cyst, external auditory canal obstruction, hearing loss, MRI, case report

## Abstract

**Purpose:** Ganglion cysts of the temporomandibular joint (TMJ) are uncommon periarticular lesions and may be diagnostically challenging because symptoms are often nonspecific. When these lesions arise posterior to the joint, they can produce otologic complaints through dynamic narrowing of the external auditory canal (EAC). Herein, we report on a histologically confirmed TMJ ganglion cyst causing position-dependent hearing loss and review the relevant literature. **Case description:** A 72-year-old woman presented with a 3-year history of bilateral preauricular pain, left-sided tinnitus, left aural fullness, and near-complete hearing loss in the left ear when the mandible was closed in occlusion. Clinical examination showed marked narrowing of the left EAC with mandibular closure. Magnetic resonance imaging demonstrated bilateral anterior disc displacement with reduction and a posterior meniscal cyst associated with the left TMJ. The lesion was excised using a preauricular approach. **Results:** Intraoperatively, the cyst was adherent to the posterior aspect of the TMJ disc and retrodiscal tissues and was noted to obstruct the EAC in the closed-mouth position. Gross examination showed a cystic structure measuring 2.4 × 2.1 × 1.0 cm which contained gelatinous material, while histopathology confirmed that the structure was a ganglion cyst. The patient’s hearing improved substantially by 4 months after surgery and had returned to normal 2 years later, with no clinical evidence of recurrence. **Conclusions:** Posterior TMJ ganglion cysts should be considered in patients with fluctuating otologic symptoms that vary with mandibular movement. MRI is valuable for diagnosis and surgical planning, and open excision can provide durable symptom resolution.

## 1. Introduction

Cystic lesions of the temporomandibular joint are rare and include both synovial and ganglion cysts [1,2,3,4,5,6,7,8,9]. Although these lesions are common in the wrist, hand, foot, and knee, they are encountered less frequently in the TMJ, where they may mimic temporomandibular disorders, parotid lesions, or otologic disease [1,2,4,5,8,10]. Ganglion cysts are pseudocysts characterized by a fibrous connective tissue wall without a true synovial lining and typically contain thick gelatinous material; in contrast, synovial cysts are true cysts lined by synovium and may communicate with the joint space [1,2,4,7,8].

The reported clinical spectrum is broad. Most patients present with preauricular swelling, pain, trismus, or nonspecific TMJ dysfunction [1,2,3,4,5,9,10]. However, because the posterior joint space is immediately adjacent to the external auditory canal, unusual otologic manifestations have also been described, including ear fullness, tinnitus, fluctuating canal masses, conductive symptoms, facial nerve palsy, trigeminal neuropathy, and intracranial extension [6,10,11,12,13,14,15]. These uncommon presentations may delay diagnosis or shift attention toward primary otologic or neurologic pathologies.

Magnetic resonance imaging is generally the most informative modality for evaluating periarticular TMJ cysts because it demonstrates the relationship of the lesion to the disc, retrodiscal tissues, and external auditory canal while also identifying associated internal derangement [1,3,4,9,10]. Nevertheless, definitive distinction between ganglion and synovial cysts remains histopathologic, and immunohistochemical work has shown that standard histology alone can misclassify a subset of lesions [7,8]. In this case report, we present a histologically confirmed ganglion cyst of the left TMJ causing dynamic EAC obstruction and position-dependent hearing loss, together with a focused review of comparable cases in the literature.

## 2. Case Report

A 72-year-old woman presented to the Oral and Maxillofacial Surgery clinic at UTHealth Houston for evaluation of temporomandibular joint pain and a peculiar left-sided auditory complaint. She described complete or near-complete hearing loss in the left ear whenever her teeth were brought into occlusion. When she opened slightly and her teeth separated, her hearing improved immediately and returned to baseline. This symptom had become increasingly bothersome over time. It was accompanied by a 3-year history of bilateral preauricular pain, greater on the left; intermittent headaches; left-sided tinnitus; and a sensation of fullness in the left ear.

Her medical history was significant for osteoarthritis, fibromyalgia, and depression. Surgical history included hysterectomy, cholecystectomy, rhytidectomy in 2008, and Mohs surgery for basal cell carcinoma of the left conchal region in 2016. Current medications included citalopram, amitriptyline, trazodone, and naproxen. She reported an allergy to doxycycline, denied tobacco use, and reported alcohol use only in social settings.

On examination, the patient was well developed, well nourished, and in no acute distress. No facial asymmetry or visible preauricular swelling was present. Bilateral preauricular scars consistent with prior facelift surgery were noted, as well as a healed scar in the left conchal region. The most striking physical finding was dynamic narrowing of the left external auditory canal: with the mandible closed in maximal intercuspation, the canal became markedly constricted and almost completely occluded; with mouth opening, the canal reopened. Intraoral examination demonstrated grossly intact dentition with mild attrition and a stable, reproducible occlusion. Maximum interincisal opening was 52 mm, although opening was associated with mild discomfort. Palpation disclosed bilateral preauricular tenderness without clicking or crepitus. Protrusive and lateral excursive movements were within normal limits, and cranial nerves V and VII were intact.

**Figure 1:**
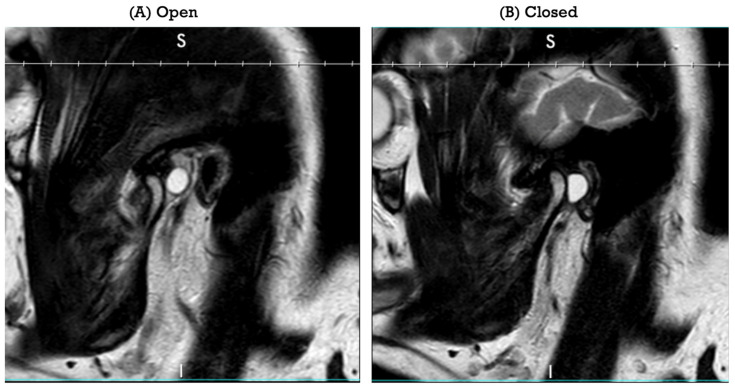
Sagittal view magnetic resonance images of the left temporomandibular joint. (**A**) View with mouth open. (**B**) View with mouth closed, demonstrating the posterior cystic lesion and its relationship to the external auditory canal. MRI sagittal views obtained with the mouth open (**left**) and closed (**right**).

Magnetic resonance imaging of the temporomandibular joints demonstrated bilateral anterior disc displacement with reduction. On the left, a posterior meniscal cyst was identified posterior to the joint, measuring approximately 7 mm in the anteroposterior dimension and 10 mm craniocaudally. The lesion was best appreciated on sagittal images obtained with the mouth open and closed and correlated clinically with dynamic EAC compromise during mandibular closure (Figure 1A,B). The imaging differential included cystic change related to internal derangement, joint effusion, and periarticular cystic lesion.

**Figure 2:**
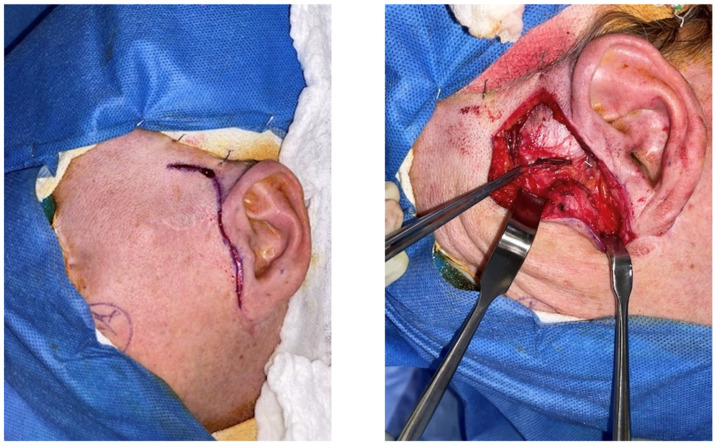
Intraoperative photographs demonstrating the preauricular approach and exposure of the temporomandibular joint region. Preauricular approach to and intraoperative exposure of the lesion.

Because the patient’s symptoms were persistent, mechanically reproducible, and strongly correlated with the identified lesion, operative excision was recommended. Under general anesthesia, a preauricular approach was utilized through a prior rhytid incision with facial nerve monitoring. After dissection through the temporoparietal fascia and exposure of the zygomatic arch and TMJ region, a cystic lesion approximately 1.5 cm in greatest operative dimension was identified adherent to the posterior aspect of the articular disc and the retrodiscal tissues (Figure 2). In the closed-mouth position, the lesion was displaced posteriorly and contributed to the obstruction of the external auditory canal. A small portion of the disc was removed en bloc with the cyst, and the external auditory canal was preserved without violation.

**Figure 3:**
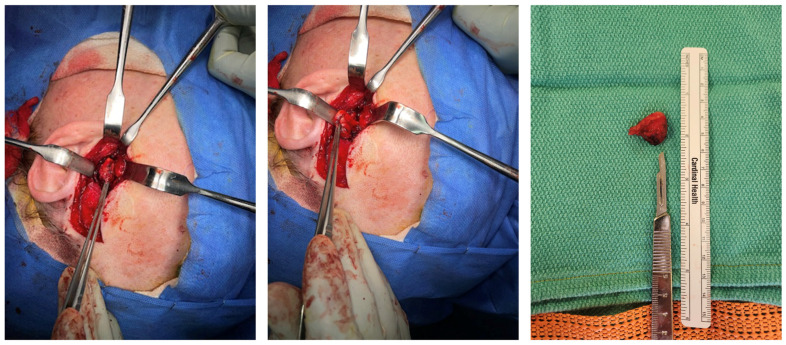
Intraoperative dissection of the cystic lesion and gross specimen after excision. Intraoperative dissection and excised cyst specimen.

**Figure 4:**
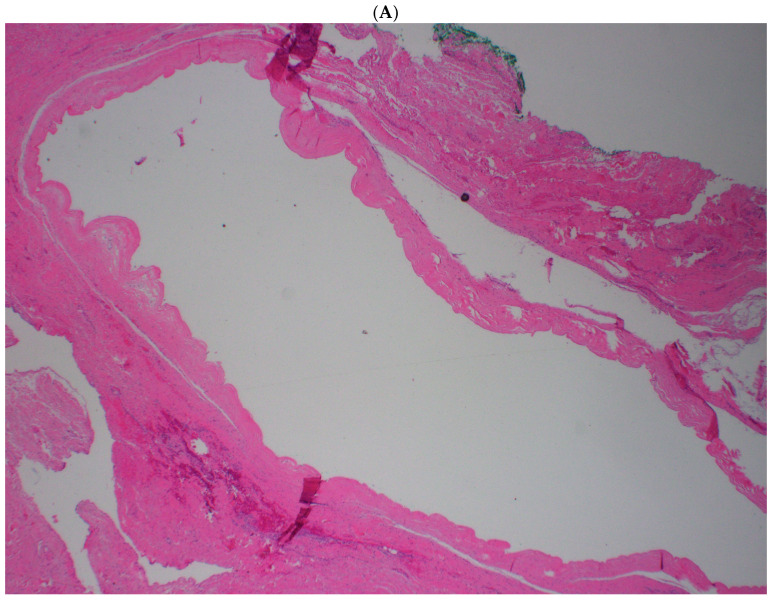

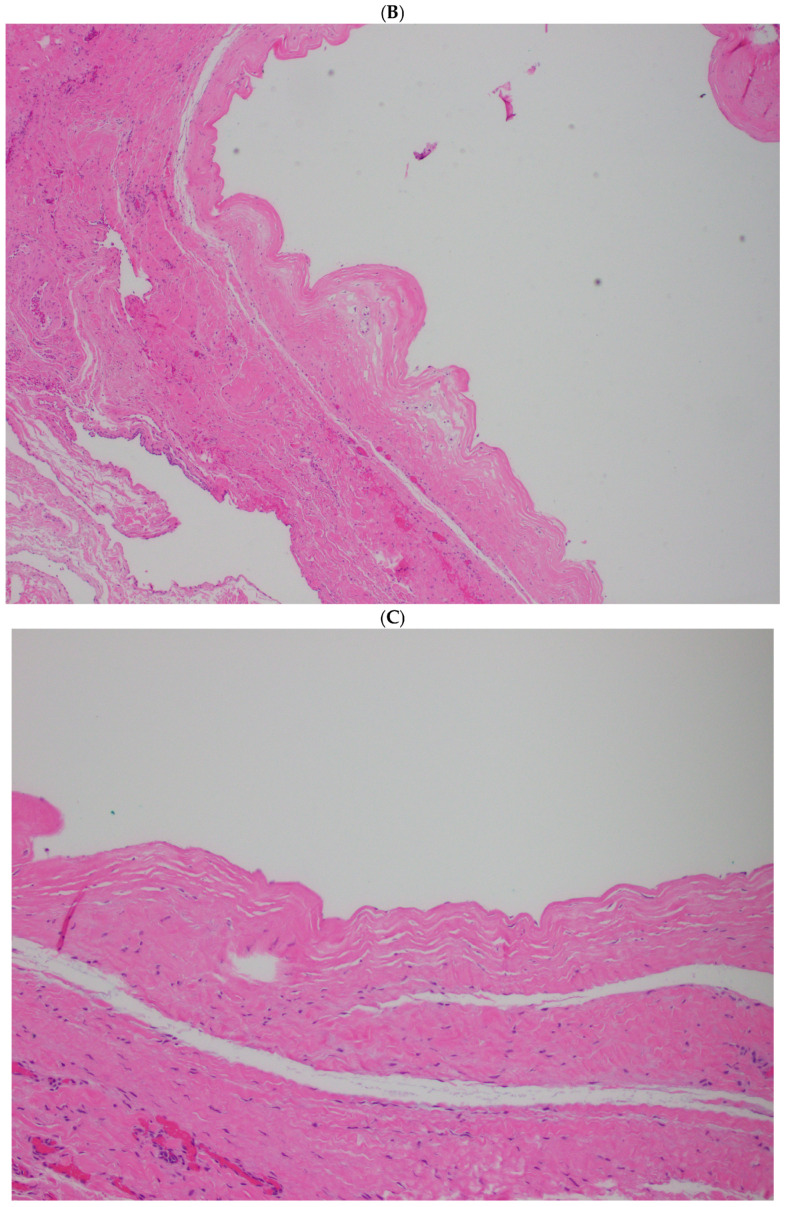
Histopathologic evaluation of the temporomandibular joint cyst. Histopathologic evaluation of the temporomandibular joint cyst. (**A**) Low-power view (×2) demonstrating a well-circumscribed cystic lesion with a fibrous connective tissue wall and a central cystic space. (**B**) Intermediate-power view (×4) showing the cyst wall architecture with a dense, undulating fibrous connective tissue lining and a smooth luminal interface. (**C**) High-power view (×10) illustrating the hypocellular fibrous cyst wall composed of dense collagen fibers without an identifiable epithelial or synovial lining.

Gross pathologic examination identified a 2.4 × 2.1 × 1.0 cm cystic structure with a homogeneous white cut surface and clear gelatinous fluid. Histopathologic examination confirmed the presence of a ganglion cyst in the left temporomandibular joint (Figure 3 and Figure 4). No epithelial or synovial lining was identified, supporting the diagnosis of a ganglion rather than a synovial cyst.

Postoperatively, the patient did well. At 4 months, she reported substantial improvement in hearing and resolution of the prior dynamic canal blockage, with both external auditory canals patent on examination. Residual symptoms were limited to mild, intermittent preauricular discomfort. At a 2-year follow-up, she reported that hearing in the left ear had returned to normal and that preauricular pain had resolved completely. No clinical recurrence was evident.

## 3. Discussion

TMJ ganglion cysts remain distinctly uncommon, and most clinicians will encounter few, if any, during training or practice [1,4,5,9]. Their rarity contributes to diagnostic delay, especially when the presenting complaint is not a visible preauricular mass. The pathogenesis is still debated, but the prevailing theory is myxoid degeneration of periarticular collagenous connective tissue caused by chronic stress, repetitive microtrauma, or degenerative change [1,4,5,9,12,13]. In contrast, synovial cysts are thought to arise from herniation or outpouching of the synovium under increased intra-articular pressure and are defined by the presence of a synovial lining [2,7]. This distinction matters both academically and pathologically, although the clinical and radiographic appearances of these cysts can be very similar.

The largest single-institution pathologic review currently available suggests that periarticular TMJ cysts may actually be more frequently synovial than ganglion when immunohistochemical methods are used to clarify the diagnosis [8]. This observation is important because the two terms are used inconsistently in older studies. From the standpoint of surgical management, however, both lesions are approached similarly when symptomatic: careful exposure, excision of the cyst wall, and preservation of adjacent structures. Histopathology remains the gold standard for definitive classification.

Our patient’s presentation was noteworthy because the dominant functional complaint was position-dependent hearing loss with mandibular closure rather than an obvious preauricular mass. Several published reports provide support for the concept that posteriorly situated TMJ cysts can manifest with otologic symptoms. Synovial cysts have been reported to cause ear pain, aural fullness, and conductive changes [2,6,7]. Ganglion cysts have also been described as fluctuant lesions or masses within the external auditory canal, associated with fullness, otorrhea, or hearing-related symptoms [11,14,15]. Eguchi et al. [10] described a ganglion cyst associated with TMJ osteoarthritis in which the external auditory canal narrowed during mouth closure, and aural fullness persisted despite treatment of the underlying joint disease. Taken together, these reports support the view that the EAC should be examined dynamically in cases of suspected posterior TMJ lesions. Selected published cases relevant to the present report are summarized in Table 1.

The dynamic nature of this patient’s complaint is anatomically plausible. With the mandible in occlusion, posterior translation and soft tissue tension likely shifted the retrodiscal cystic mass toward the anterior canal wall, functionally narrowing the EAC. When the mouth opened, the joint relationship changed, and the canal lumen reopened. This mechanical explanation fits both the clinical examination and the MRI appearance. Importantly, it also helps differentiate the lesion from primary middle ear disease, patulous eustachian tube symptoms, and other otologic causes of fluctuating hearing complaints.

A limitation of this report is the absence of formal preoperative and postoperative audiometric testing. However, the patient’s symptoms were reproducible with mandibular position, correlated with clinical and MRI evidence of dynamic external auditory canal narrowing, and resolved after surgical excision, supporting the proposed mechanism.

Imaging played a central role in diagnosis and operative planning. MRI is useful because it shows the relationship of the lesion to the disc, retrodiscal tissues, and surrounding soft tissues, and helps identify any coexisting internal derangement [1,3,4,9,10]. CT can be helpful when bony erosion or external auditory canal involvement is suspected, as reported in several otology-oriented case reports [11,14,15]. In the present case, MRI was sufficient to show bilateral reducible anterior disc displacement and a posterior cystic lesion on the left that corresponded precisely to the patient’s symptoms.

The differential diagnosis of a periarticular TMJ cystic lesion includes synovial cysts, ganglion cysts, parotid lesions, inflammatory pseudocysts, synovial chondromatosis, and, less commonly, neoplastic processes. When ear symptoms dominate the presentation, exostoses, osteoma, canal cholesteatoma, inflammatory canal lesions, and skull-base pathology can also be included [14,15]. In our case, histopathology excluded these alternatives by demonstrating fibrous connective tissue with gelatinous contents and no true epithelial or synovial lining.

Open excision via a preauricular approach remains the most commonly reported treatment for symptomatic TMJ ganglion cysts [1,4,5,9,10]. This approach provides excellent access to the lateral and posterior joint region and allows for simultaneous management of adherent capsular or discal tissue. Favorable outcomes are typical when the cyst is completely removed, although recurrence has been described, particularly after incomplete excision or in the context of persistent joint pathology [9]. In our patient, excision resulted in early improvement and durable resolution of hearing-related symptoms through 2 years of follow-up.

This case adds to the limited literature on posterior TMJ ganglion cysts with otologic manifestations and further emphasizes that the clinician should not ignore symptoms that change with mandibular movement. Dynamic external auditory canal narrowing during clinical examination is a particularly useful clue. For OMFS surgeons, otolaryngologists, and radiologists, correlating the patient’s symptom pattern with careful physical examination and cross-sectional imaging can enable timely diagnosis and definitive treatment.

## 4. Conclusions

Temporomandibular joint ganglion cysts are rare lesions, but they should be considered when patients present with fluctuating otologic symptoms that vary with mandibular position. In the present case, a posterior TMJ ganglion cyst produced dynamic external auditory canal obstruction and reproducible hearing loss when the mandible was closed.

MRI was valuable when defining the lesion and its relationship to the joint and external auditory canal. Open excision through a preauricular approach provided definitive diagnosis and durable symptom relief. Awareness of this uncommon presentation may prevent delayed diagnosis and unnecessary treatment for presumed primary otologic disease.

## Figures and Tables

**Table 1:** Selected published reports of synovial and ganglion TMJ cysts relevant to the present case.

Study	Year	Lesion	Key Presentation	Management	Outcome/Note
Deng et al. [5]	2010	Ganglion	Preauricular swelling; condylar erosion on CT	Preauricular excision	No recurrence at 24 months
Spinzia et al. [2]	2011	Synovial	Right preauricular swelling	Surgical excision	Histologically confirmed synovial cyst, no recurrence at 18 months
Mumert et al. [12]	2012	Ganglion	Facial nerve palsy; intracranial extension	Surgical management	Illustrates atypical neurologic presentation
Savolainen and Kellokoski [4]	2013	Ganglion	Painful prominence; altered sensation	Preauricular excision	No recurrence at 6 months
Vera-Sirera et al. [7]	2013	Synovial	Pathologic characterization	Excision/IHC analysis	Highlighted diagnostic value of immunohistochemistry
Verma and Chambers [3]	2015	Synovial	Painful preauricular swelling	Surgical excision	Long-term follow-up reported
Partridge et al. [8]	2016	Mixed cohort	Largest single-institution TMJ cyst series	Retrospective review	TMJ periarticular cysts more often synovial than ganglion
Buckley et al. [6]	2019	Synovial	Bilateral TMJ cysts	Surgical management	Bilateral occurrence is exceptionally rare
Nys et al. [9]	2020	Mixed case series	Pain and/or progressive unilateral preauricular swelling	Case-based management	Emphasized recurrence risk and spectrum of symptoms
Eguchi et al. [10]	2020	Ganglion	Trismus, pain, aural fullness; EAC narrowing with mouth closure	Surgery after TMJ workup	Supports dynamic canal narrowing mechanism
Esmaili et al. [11]	2018	Ganglion	Fluctuating cystic lesion within EAC	Observation	Asymptomatic canal lesion can be surveilled
Present case	2026	Ganglion	Position-dependent hearing loss with EAC obstruction	Preauricular excision	Complete symptom resolution at 2 years

## Data Availability

The data presented in this study are available upon request from the corresponding author.
